# Cost-effective interventions to prevent non-communicable diseases: increasing the evidence base in India and other low- and middle-income settings

**DOI:** 10.1186/s12916-020-01850-0

**Published:** 2020-12-09

**Authors:** Karen Eggleston, Radhika Jain

**Affiliations:** Freeman Spogli Institute for International Studies (FSI), Asia Health Policy Program, Asia-Pacific Research Center (APARC), Stanford University, Stanford, USA

**Keywords:** Cost-effectiveness, Diabetes, Non-communicable diseases, India

## Background

India, as part of its bid to achieve universal health coverage, has expanded government health programs over the last two decades, most notably with the establishment of the National Health Mission and the rollout of public health insurance programs targeting poor households [1]. However, national spending on health remains among the lowest in the world. As the government increasingly takes on the role of purchaser of health care, decisions about the allocation of scarce resources for health will have substantial fiscal and health consequences and must be based on evidence. Additionally, in order to control costs and effectively address the growing chronic disease burden, public programs will need to find ways to integrate curative hospital services with the most cost-effective preventive and primary interventions. Currently, in part because the evidence base on economic evaluations of health interventions in India remains sparse and of low quality [2], decisions about which health care services to cover are typically made by expert committees rather than through systematic assessments of efficacy and cost-effectiveness. However, in recent years, the government has taken several steps towards establishing the infrastructure for evidence-based priority setting and resource allocation [3], including the establishment of a body for Health Technology Assessment in India (HTAIn) within the Department of Health Research to collate and generate evidence on the clinical efficacy and cost-effectiveness of new and existing health technologies and programs [4]. Research evidence on the cost-effectiveness of both preventive and curative health interventions in the Indian context is going to be a critical input to the HTAIn.

## Evidence of cost-effective prevention of diabetes and non-communicable disease

Chronic non-communicable diseases (NCDs) affect more than 20% of the Indian population [5], with incidence and prevalence projected to increase substantially as the population aged 60 and over increases. Levels of several critical risk behaviors, such as alcohol and tobacco use, low physical activity, and unhealthy diet are increasing in socioeconomic status and will require explicit intervention beyond economic development or access to curative care alone. Because the risk factors for chronic diseases are overlapping, the benefits of preventive interventions targeting them are likely to extend beyond preventing diabetes or any other single NCD. Numerous reviews find that population-based interventions, such as advertising bans, food industry regulations, mass media campaigns, and tobacco and alcohol taxation are most cost-effective due to their very low marginal costs and high coverage [5–7]. However, these interventions require concerted public and political effort and have not been scaled up in India to date. Targeted individual or community level preventive interventions that can be implemented at a more local level may be a promising and feasible complement to population interventions. Lifestyle modification to reduce weight, increase activity, and improve diets and metformin to prevent diabetes have been found to be highly cost-effective in the Indian context, although universal diabetes screening is not [8].

The cost-effectiveness study of the Kerala Diabetes Prevention program (K-DPP) by Sathish and colleagues adds evidence on how to prevent diabetes cost-effectively in India and other low- and middle-income countries (LMIC) [9]. Several features of the study are worth highlighting. The authors present a cost-effectiveness analysis of 1007 participants in the K-DPP, finding the societal cost per QALY gained was US$155, and the health system cost per QALY gained about one third of that (i.e., $US50). The corresponding estimates of cost per diabetes case prevented were almost twice as high, based on an absolute risk reduction of 2.1% that was not statistically significant. Their estimates suggest that K-DPP was cost-effective. More precisely, the uncertainty analyses suggest that 80% or more of bootstrap estimates were cost-effective and that the ICERs remained below the cost-effectiveness threshold in sensitivity analyses moving the costs and effectiveness up or down by 10–30%. Unsurprisingly for just a 2-year period, results are not sensitive to differences in discounting of costs and effects.

Of course, no study is without some limitations, and the authors appropriately acknowledge a long list. Sensitivity analyses at the most extreme reduced the point estimate of effectiveness by 30%, rather than the 100% reduction that would be implied by effectiveness being statistically insignificantly different from zero. More generally, the effectiveness and cost effectiveness of interventions like the one studied are likely to be sensitive to the study population and duration. This study covers mostly poor unskilled workers in one rural sub-district of India for 2 years. Benefits of preventive care typically accumulate over time, which could increase the cost effectiveness of the intervention over a longer time horizon. On the other hand, it is unclear to what extent the effects of one-time behavior change interventions will be sustained rather than decay over time. Furthermore, different populations may have different levels of take-up of the intervention, and effectiveness, conditional on take-up, may vary across a range of factors, such as access to outside sources of the same information provided in the intervention, or baseline health status. Costs may vary across populations with different preferences and opportunity costs. There may also be economies of scale. A larger sample over a longer time horizon is needed to clarify these dimensions of cost-effectiveness.

Nevertheless, the study shows potential cost-effectiveness in “nudging” the participants towards a healthier lifestyle, through suggestive reductions in tobacco and alcohol use and waist circumference. The results highlight the importance of continued research on community-based promotion of healthy lifestyles. After all, many health conditions could be prevented if all middle-aged individuals adhered to lifestyles with high physical activity, healthy eating habits, no tobacco, limited alcohol, and adequate sleep—the risk factors targeted in K-DPP. Moreover, such health-promoting interventions complement existing policy efforts to support healthy aging [10].

## Conclusions

While the analysis by Sathish and colleagues provides an excellent first step [9], future studies covering larger and more representative populations over a longer time period—as are already underway—remain important for more generalizable assessments to inform policy decisions.
